# A deep learning-based automated algorithm for labeling coronary arteries in computed tomography angiography images

**DOI:** 10.1186/s12911-023-02332-y

**Published:** 2023-11-06

**Authors:** Pengling Ren, Yi He, Ning Guo, Nan Luo, Fang Li, Zhenchang Wang, Zhenghan Yang

**Affiliations:** 1 Department of Radiology, Beijing Friendship Hospital, Capital Medical University, No. 95 Yongan Road, Xicheng District, Beijing, 100050 P.R. China; 2 Shukun (Beijing) Technology Company Ltd, Beijing, P.R. China

**Keywords:** Coronary artery, Segments, Automatic labeling, Computed tomography angiography, Deep learning

## Abstract

**Objective:**

Using two three-dimensional U-Net architectures for myocardium structure extraction and a distance transformation algorithm specifically for the left circumflex artery, we have designed a fully automated algorithm for coronary artery labeling in coronary computed tomography angiography (CCTA) images.

**Methods:**

In this retrospective analysis, a cohort of 157 patients who had undergone coronary computed tomography angiography (CCTA) was included. An automated coronary artery labeling algorithm was developed using a distance transformation approach to delineate the anatomical segments along the centerlines extracted from the CCTA images. A total of 16 segments were successfully identified and labeled. The algorithm’s outcomes were recorded and reviewed by three experts, and the performance of segment detection and labeling was assessed. Additionally, the level of agreement in manually labeled segments between two experts was quantified.

**Results:**

When comparing the labels generated by the experts with those produced by the algorithm, it was necessary to modify or eliminate 117 labels (5.4%) out of 2180 segments assigned by the algorithm. The overall accuracy for label presence was 96.2%, with an average overlap of 94.0% between the expert reference and algorithm-generated labels. Furthermore, the average agreement rate between the two experts stood at 95.0%.

**Conclusions:**

Based on the labels of the clinical experts, the proposed deep learning algorithm exhibits high accuracy for automatic labeling. Therefore, our proposed method exhibits promising results for the automatic labeling of the coronary arteries and will alleviate the burden on radiologists in the near future.

## Introduction

Coronary computed tomography angiography (CCTA) is a widely employed diagnostic tool for cardiovascular diseases [1]. In clinical practice, radiologists and cardiologists traditionally engage in a manual reconstruction process using two-dimensional transaxial images before compiling the radiology report. Following the imaging guidelines established by the Society of Cardiovascular Computed Tomography (SCCT), radiologists and cardiologists are required to document the pathological findings of individual arteries or segments as determined through this reconstruction process [2–4]; however, this process is tedious. Furthermore, in clinical settings, during the analysis of CCTA images, extracting the centerlines of the coronary arteries to obtain noninvasive information on the cardiovascular system is a vital first step, particularly when treating individuals with suspected coronary artery disease [5]. However, manually extracting the centerlines of the coronary arteries is tedious and laborious; therefore, this process is not suitable in clinical settings. To resolve these concerns, automated labeling methods have been applied to extract coronary artery centerlines and label them. Automatic coronary artery labeling approach does not intend to replace physicians, but rather to hasten the preparation and generation of the patient’s report. Thus, it is important to develop an accurate automatic labeling algorithm because coronary artery labeling forms the basis for diagnosing coronary artery disease.

Recently, several methods for coronary artery labeling have been developed [6–12]. Gulsun and colleagues employed topological and geometric data from the coronary tree to establish the relationship between nodes within a labeled model and unmarked datasets [8]. Furthermore, Guanyu et al. used a statistical coronary tree model to develop a two-step method. Initially, the identification of the four primary branches involved their alignment with the coronary tree model. Subsequently, the labeling of the segments in both the four main branches and side branches was determined based on clinical criteria [10]. Qing et al. devised a labeling algorithm that assessed matching costs among segments within each subtree, aiming to establish an optimal correlation between the model and the patient tree, utilizing three-dimensional (3D) models [7]. Additionally, TreeLab-Net was developed by Dan et al., combining a multilayer perceptron encoder network with bidirectional tree-structured long–short-term memory [9]. Furthermore, Akinyemi et al. introduced a method with two key phases: (1) training a multivariate Gaussian classifier using labeled anatomy to calculate mean vectors and a covariance matrix for each anatomical class, pooling them over all classes with a set of features, and (2) generating all possible label combinations for each test anatomy based on a series of topological and geometric rules [6]. Nonetheless, it’s important to note that variations exist in the reported results of these CCTA-based labeling techniques, with overall accuracy ranging from 85.0 to 92.0%.

All investigations into the automated labeling of CCTA images have consistently demonstrated high levels of accuracy, typically ranging from 87 to 100.0%, when it comes to the three principal arteries: the right coronary artery (RCA), left anterior descending artery (LAD), and left main coronary artery (LM). However, studies have noted that accuracy levels for the left circumflex artery (LCx) and its associated side branches tend to be relatively lower [4, 13], primarily due to notable vascular variations. Additionally, distinguishing between LAD and LCx using deep learning techniques and simple geometric rules can be a challenging task. Furthermore, the intricate nature of vascular structures presents formidable hurdles in the automated labeling of the entire vessel tree [14, 15]. Therefore, the development of an accurate automated labeling method is imperative to streamline the labeling process for both the principal arteries and their side branches, including LCx and the first and second obtuse marginal branches (OM1 and OM2).

In the present study, we have presented an efficient automatic labeling algorithm for coronary anatomy by using a distance transformation algorithm and involving experts to evaluate the automatic identification results, as well as analyzed the consistency in coronary artery labeling among the experts.

## Methods

### Patient data

This study received approval from the Research Ethics Board of our institution, and the institutional review board granted a waiver for informed consent. The study included retrospectively collected CCTA scans from 157 patients acquired between June 20, 2017, and May 12, 2019. Patients who had undergone cardiac procedures such as bypass surgery or percutaneous coronary intervention were excluded. You can find the baseline characteristics of these 157 patients in Table 1. It is important to note that all patients exhibited right coronary artery dominance.

**Table 1:** Characteristics of the study patients

Characteristic (n = 157)	Value
Age	66(59,71)
Male gender	102(64.6%)
Height (cm)	166.38 ± 9.97
Weight (kg)	72.2 ± 11.14
BMI (kg/m^2^)	26.4 ± 7.46
Heart rate (bpm)	66.94 ± 17.15
Smoker (%)	78(49.4%)
Hypertension (%)	109(69.0%)
Hyperlipaemia (%)	91(57.6%)
Diabetes (%)	68(43.0%)
Family history of coronary heart disease (%)	15(9.5%)
Other past medical history (%)	31(19.6%)

### CCTA acquisition

A 64-row detector CT system (LightSpeed CT, GE Healthcare, US), 128-row detector CT system (Brilliance CT, Philips Medical Systems, The Netherlands), and 256-row detector computed tomography (CT) system (Revolution CT, GE Healthcare, US) were used for data acquisition. Furthermore, a prospective electrocardiography-triggered CCTA with a 0.625-mm-thick slice. For body weights of < 100 kg, iodinated contrast medium (either iopromide 370 or iohexol 350) was injected at the rate of 5 mL/s; on the other hand, for body weights of ≥ 100 kg, the medium was injected at a 6 mL/s rate. Thereafter, saline chaser bolus (50 mL) was administered.

### Automatic labeling technique

**Fig. 1:**
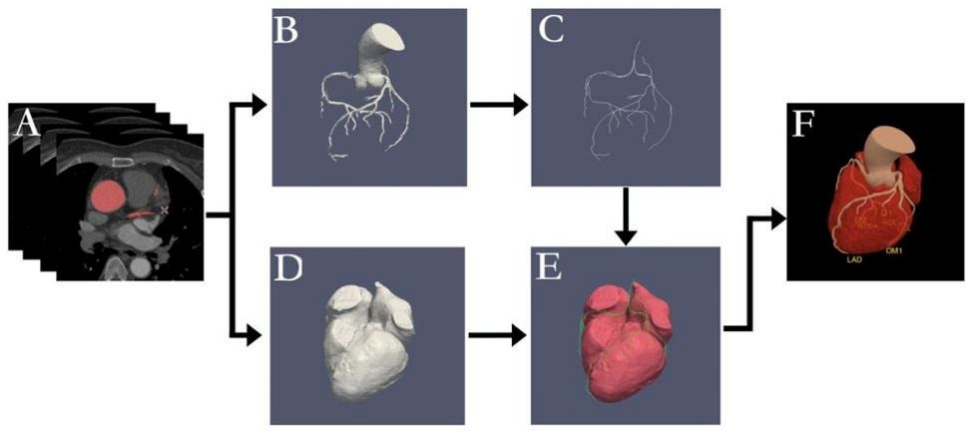
Illustration of the steps involved in the identification of all coronary artery segments. (**A**) Obtaining the CCTA image set; (**B**) Extracting the 3D volume of the coronary arteries; (**C**) Extracting the centerlines of the coronary arteries; (**D**) Extracting the myocardium structure and 3D volume data based on a 3D-U-Net-based deep learning model; (**E**) Labeling and segmenting the centerlines of the LM, RCA, LAD, and LCx with the spatial structure rule or distance transformation method; (**F**) Assembling the results and visualizations for diagnosis

Figure 1 illustrates the overview of the framework. In this study, three sequential steps were included based on the processing pipeline: (1) Centerline extraction from the segmented coronary arteries. (2) Extraction of the myocardial structure and acquisition of 3D volume data. (3) Labeling of the artery branches using distance measurements relative to the myocardium.

#### Centerline extraction

This algorithm depends on the centerline of the coronary arteries [13, 16, 17]. After obtaining the CCTA image set, coronary artery extraction was performed using the 3D U-Net model described previously [18]. This convolutional neural network model operates on 3D images and is trained on a large annotated 3D image dataset to predict segmentations. It comprises a U-shaped network with an encoding path that captures circumstances and a symmetric decoding path for precise localization. This method is particularly effective in tasks such as ours, typically achieving Dice coefficient values of 85–95%. For the extraction of the centerlines of the coronary arteries, a traditional skeletonization algorithm was used. This algorithm uses iterative erosion but preserves the topological structure until the shape is reduced to a skeleton. Iterative erosion is a morphological image processing technique involving the repeated application of an erosion operation to an image. Erosion is a fundamental operation in the processing of morphological images that removes small objects or features from an image by shrinking the boundaries of the foreground regions. In iterative erosion, the erosion operation is repeatedly applied to the image until a desired level of feature removal is achieved. In particular, this method is useful for removing small objects or features embedded within larger structures or regions of interest. The iterative nature of the technique helps fine-tune the amount of feature removal because the number of iterations can be adjusted to achieve the desired level of erosion. Furthermore, this method can remove small blood vessels or other structures that may interfere with the detection or segmentation of larger structures, including organs.

In this study, skeletonization was performed to transform the binary 3D representation of the segmented arteries into a 1-pixel-wide centerline structure that retains the general shape of the arteries; this provided an accurate depiction of their centerlines. We applied spatial structure rules or the minimum 3D distance transformation method to label and segment the centerlines of the left main coronary artery (LM), right coronary artery (RCA), left anterior descending artery (LAD), and left circumflex artery (LCx). Subsequently, the outcomes were compiled and visualized for diagnostic purposes (refer to Fig. 1). The centerlines of each artery were stored in separate VTK files, preserving the 3D coordinates of each point along the centerlines, with an index denoting the sequence of these points. The coordinate system had its positive directions aligned with the right-to-left, back-to-front, and top-to-bottom orientations for the x, y, and z-axes, respectively.

#### Extraction of the myocardium structure

**Fig. 2:**
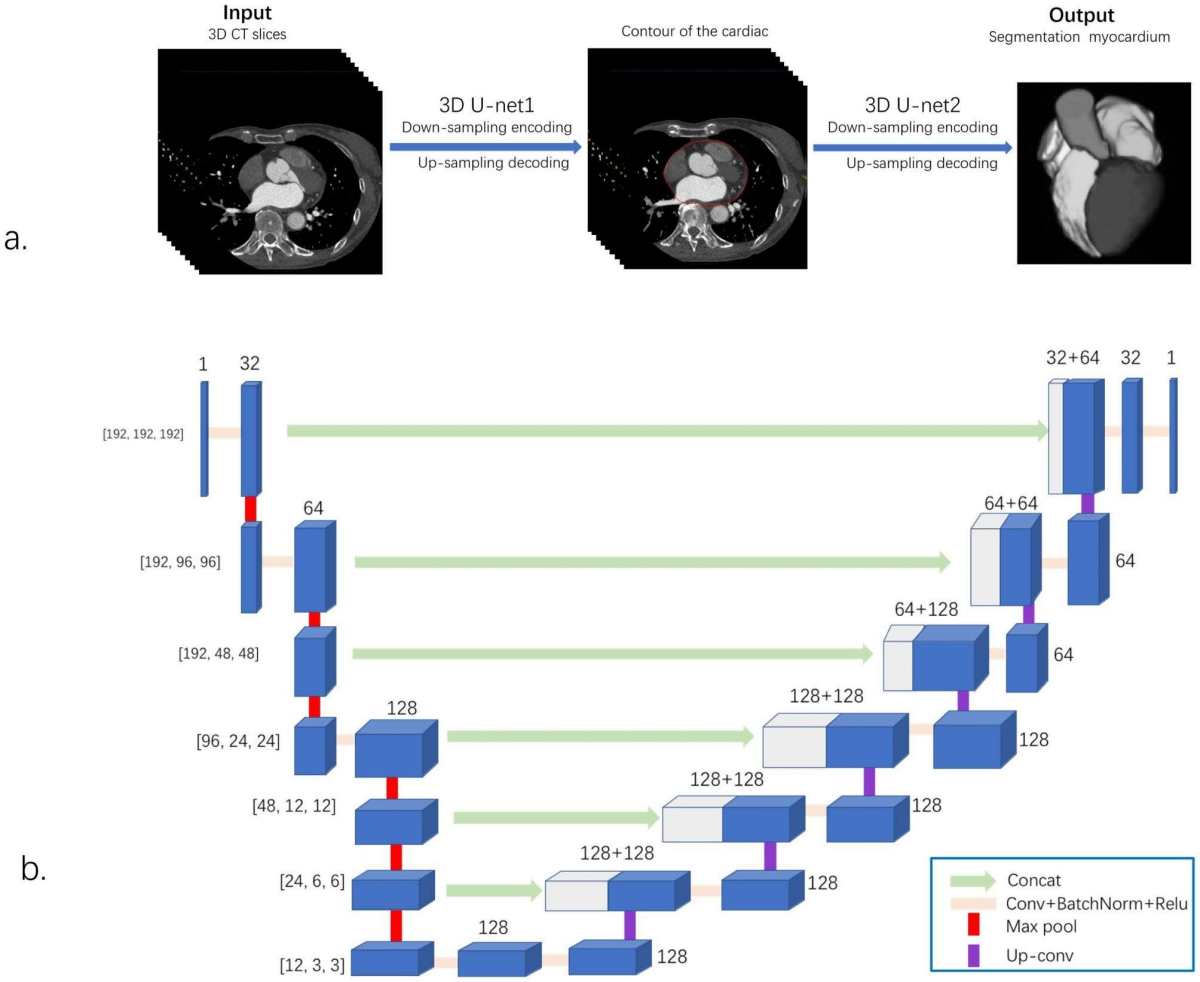
Automatic segmentation frame-work for myocardium structure and 3D volume data extraction. (**a**) The entire myocardium structure segmentation pipeline. The myocardium structure segmentation and extraction are automatically completed after two 3D U-Nets. (**b**) Schema and structure of the 3D U-Net architecture

The algorithm served to acquire the coordinates for each point located on the myocardium’s surface. Within this process, an automated segmentation framework was established, employing a pair of 3D U-Net architectures to extract both the myocardial structure and 3D volume data. Figure 2 demonstrates that the network adopts a typical encoder–decoder structure and replaces the traditional 2D convolution with 3D convolution via 3D convolutions, up-convolutional layers, and max pooling. The encoder comprises multiple convolutional and pooling layers. The convolution layer uses a 3D convolution kernel as well as a rectified linear unit and batch normalization for activation and normalization, respectively. Subsequently, spatial down sampling was achieved using a 3D max pooling layer; this gradually decreased the spatial resolution of the feature map so as to extract higher-level features. The decoder comprises several convolutional and upsampling layers. In each decoder layer, a skip connection is introduced to connect the corresponding layer feature map of the encoder with the decoder [19]. Class imbalance, where the scarcity of positive cases often leads to the development of biased models that are more accurate in predicting negative cases, is a common challenge in clinical image analysis. To tackle this concern, techniques for image augmentation, including flipping, rotation, and scaling, were employed to enhance the diversity of the training data and enhance the model’s performance.

During the preprocessing phase, the slight tilt of the organs was rectified via a rigid transformation involving rotation and translation operations. This correction ensured that the position and angle of the organs in the image were accurately adjusted. Furthermore, using standard methods described in our previous study, encompassing rescaling, windowing, and noise reduction, the images were further preprocessed [20]. In addition, cropping was conducted to concentrate on the region of interest and minimize computational requirements. Detailed specifications are summarized in our previous study. The preprocessed CCTA images were used as the input for the first U-net model, generating heart contour coordinates as an output. Subsequently, the second U-net model was adopted for fine-tuning the segmentation of the myocardium. We used 482 epochs and a batch size of 32 as the model hyperparameters.

#### Distance transformation algorithm for LCx

In this research, we categorized and assigned labels to a total of 16 coronary artery segments, including the proximal (p), middle (m), and distal (d) sections of the right coronary artery (RCA), left anterior descending artery (LAD), and the proximal, middle, and distal sections of the left circumflex artery (LCx). Additionally, labels were applied to the right posterior descending artery (R-PDA), left main coronary artery (LM), right posterior lateral branch (R-PLB), ramus intermedius (RI), obtuse marginal branches 1 and 2 (OM1 and OM2), and the first and second diagonal branches (D1 and D2). The labeling methodology employed for the identification of RCA, R-PLB, R-PDA, LM, LAD, and their respective side branches followed the procedures outlined by Chenjun et al. [21]. Meanwhile, the LCx identification process was based on myocardium distance.

From an anatomical standpoint, the left circumflex artery (LCx) courses through the left atrioventricular groove, situated between the left ventricle (LV) and the left atrium (LA). On the other hand, the left anterior descending artery (LAD) is typically situated within the epicardial fat of the anterior interventricular septum. While there are occasional variations in artery anatomy, it is generally observed that the distances from the LA and LV to the LCx are consistently smaller than those to the LAD. Leveraging this anatomical characteristic, a distance transformation method was developed for the identification of the LCx. The labeling process was as follows:

1. The LCx extends positively along the y-axis and negatively along the x-axis. Utilizing this characteristic, we sequentially stored potential LCx candidates in an array termed “LCx_possible_list.“ The initial point of these candidate LCxs coincided with the starting point of the LAD, marking the endpoint of the LM.
2. Employing a modified distance transformation technique, we computed the distances from the centerline points of the potential LCx candidates to both the LA and LV as reference distances. Each point on the LCxs was represented by coordinates [x, y, z], while the coordinate sets for the LA and LV were denoted as A ([x1, y1, z1], [x2, y2, z2], [x3, y3, z3]…[xi, yi, zi]) and B ([x1, y1, z1], [x2, y2, z2], [x3, y3, z3]…[xj, yj, zj]), respectively. Subsequently, distances were calculated using Eqs. 1 and 2, with DminLA and DminLV representing the distances between these LCx points and the LA and LV, respectively.

1 $${D_{minLA}} = \mathop {{\rm{min}}}\limits_{\left( {{x_{i,}}\,{y_{i,}}\,{z_{i,}}} \right)} \mathop {}\limits_{ \in {\rm{A}}} \sqrt {{{(x - {x_i})}^2} + {{\left( {y - {y_i}} \right)}^2} + {{\left( {z - {z_i}} \right)}^2}} $$

2 $${D_{minLV}} = \mathop {{\rm{min}}}\limits_{\left( {{x_{i,}}\,{y_{i,}}\,{z_{i,}}} \right)} \mathop {}\limits_{ \in B} \sqrt {{{(x - {x_j})}^2} + {{\left( {y - {y_j}} \right)}^2} + {{\left( {z - {z_j}} \right)}^2}} $$

3) In the context of each potential LCx candidate, we recorded the number of points with reference distances less than 25 mm to the LA and those with reference distances less than 30 mm to the LV as unique attributes.
4) The combined total of these two attributes for each centerline indicated the artery’s proximity to both the LA and LV. The candidate LCx with the highest cumulative value was chosen as the preferred option. Lastly, the LCx with the longest centerline length was established as the final selection.

Python 2.0 was used to complete the entire automatic labeling algorithm. In each case, based on the centerline, 16 segments were identified. After completing the automatic labeling process, the results were reviewed by experts based on the SCCT label standards and judged for accuracy.

### Evaluation measures

The labeling outcomes underwent independent assessment by two experts, each possessing a minimum of 4 years of experience in cardiac CT imaging. Labels were manually corrected in cases of absence or inaccuracy. In the event of disagreements between these two experts, a third expert with over 10 years of experience in cardiac CT imaging was consulted to make the final determination.

For each segment, both presence and overlap were subject to evaluation. Additionally, the agreement percentage between the two experts for each section was computed. To assess the performance of the automated labeling algorithm, a confusion matrix was employed. To maintain objectivity, the experts remained unaware of clinical histories and patient identities.

#### Presence

Since the automatic labeling algorithm might omit or inaccurately assign labels to certain segments, an assessment was conducted to determine the presence or absence of each of the 16 segments. True positives (TP) were segments labeled by both the algorithm and the experts, while false negatives (FN) were segments labeled by the experts but not by the algorithm. False positives (FP) represented segments labeled by the algorithm but not by the experts, and true negatives (TN) were segments not labeled by both the algorithm and the experts. Precision, sensitivity, accuracy, and F1 scores were computed to assess the segment detection performance for each segment.“

#### Overlap

After confirming the presence of labels, the starting and ending points of a labeled segment, determined through the automatic labeling method, underwent initial evaluation by the two experts. Subsequently, they assessed whether the label for the segment was accurate and recorded any necessary corrections.

For each label, if both experts concurred with the results produced by the automatic labeling algorithm, the automatically assigned label was considered correct. However, if there was a lack of agreement between the two experts regarding the results, the automatically assigned label was deemed incorrect. In cases where expert 1’s judgment differed from that of expert 2, the final decision was deferred to expert 3. Overlap was quantitatively measured to assess the accuracy of segment labeling, a method also employed in previous studies [8, 18]. The confusion matrix was utilized to provide insights into the performance of the automatic labeling algorithm.

### Statistical analysis

The performance evaluation outcomes regarding the presence of a segment, which encompassed precision, sensitivity, accuracy, and F1 scores, were reported as numerical values. The level of agreement or disagreement pertaining to the presence of a segment was expressed as a percentage. Additionally, overlap accuracy was provided as **a numerical value.**

## Results

Automatic labeling of the coronary tree was executed on a personal computer equipped with a Xeon Silver 2.2 GHz processor and 64 GB RAM. The total number of segments subjected to labeling by both the algorithm and experts was 2148.

Table 2 displays the comprehensive findings. The overall accuracy for segment presence was 96.2%. Specifically, labels for three main arteries, namely, RCA, LAD, and LM, consistently achieved a 100.0% accuracy rate. Additionally, the labeling accuracy for pCx and LCx segments was notably high, at 99.4% and 96.2%, respectively. However, the labeling accuracy was relatively lower for R-PLB, D2, and RI segments, with respective accuracy rates of 79.0%, 88.5%, and 84.7%, in comparison to the three primary arteries.

**Table 2:** Evaluation of whether the segment was present, including precision, sensitivity, accuracy, and F1 score

Segment	TP	TN	FP	FN	Precision	Recall	Accuracy	F1
pRCA	157	0	0	0	100.0%	100.0%	100.0%	100.0%
mRCA	157	0	0	0	100.0%	100.0%	100.0%	100.0%
dRCA	156	1	0	0	100.0%	100.0%	100.0%	100.0%
R-PDA	139	16	1	1	99.3%	99.3%	98.7%	99.3%
R-PLB	94	30	0	33	100.0%	74.0%	79.0%	85.1%
LM	157	0	0	0	100.0%	100.0%	100.0%	100.0%
pLAD	157	0	0	0	100.0%	100.0%	100.0%	100.0%
mLAD	157	0	0	0	100.0%	100.0%	100.0%	100.0%
dLAD	156	0	1	0	99.4%	100.0%	99.4%	99.7%
D1	152	3	0	2	100.0%	98.7%	98.7%	99.4%
D2	110	29	12	6	90.2%	94.8%	88.5%	92.4%
pCx	156	0	0	1	100.0%	99.4%	99.4%	99.7%
LCx	151	0	0	6	100.0%	96.2%	96.2%	98.1%
OM1	128	19	1	9	99.2%	93.4%	93.6%	96.2%
OM2	75	72	6	4	92.6%	94.9%	93.6%	93.8%
RI	46	87	11	13	80.7%	78.0%	84.7%	79.3%
In total	2148	257	32	75	98.5%	96.6%	95.7%	97.6%

**Fig. 3:**
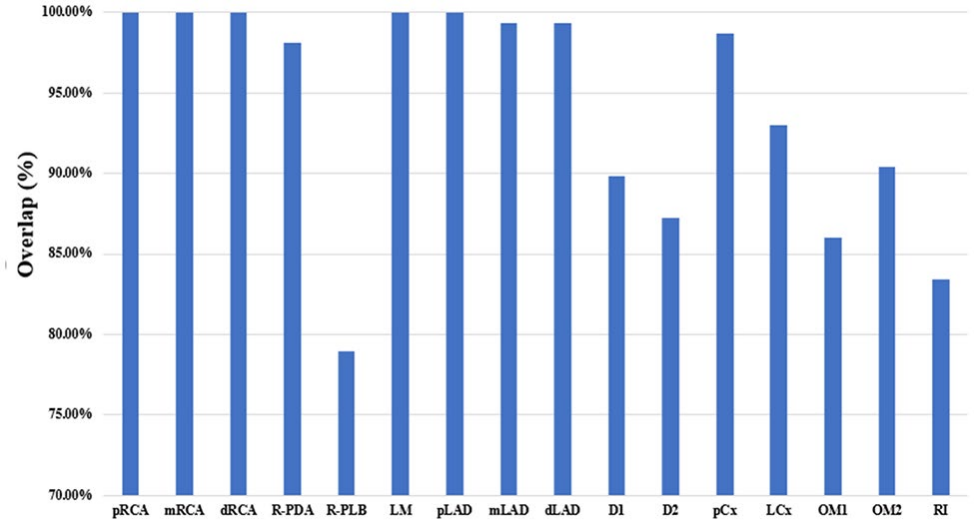
Overall overlap results for each segment

**Fig. 4:**
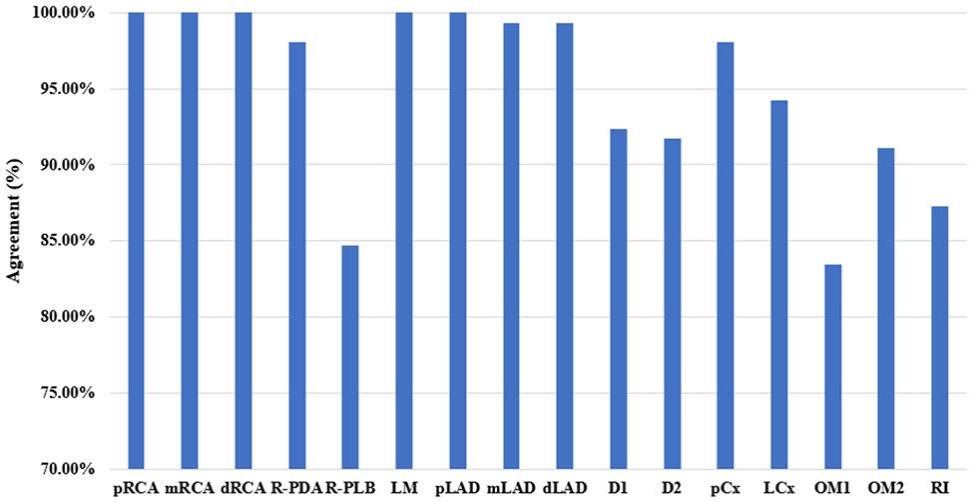
An agreement between expert 1 and expert 2

Figure 3 depicts the results of overlap accuracy determination. All labels exhibited overlaps exceeding 78.0% with pRCA, mRCA, dRCA, and pLAD. Remarkably, LM exhibited a 100.0% overlap. Moreover, pCx and LCx displayed high accuracy in overlap, achieving rates of 98.7% and 93.0%, respectively. After averaging the overlap discrepancies between the experts, the average overlap accuracy for labeling the 16 segments stood at 94.0% (as depicted in Fig. 4).

**Fig. 5:**
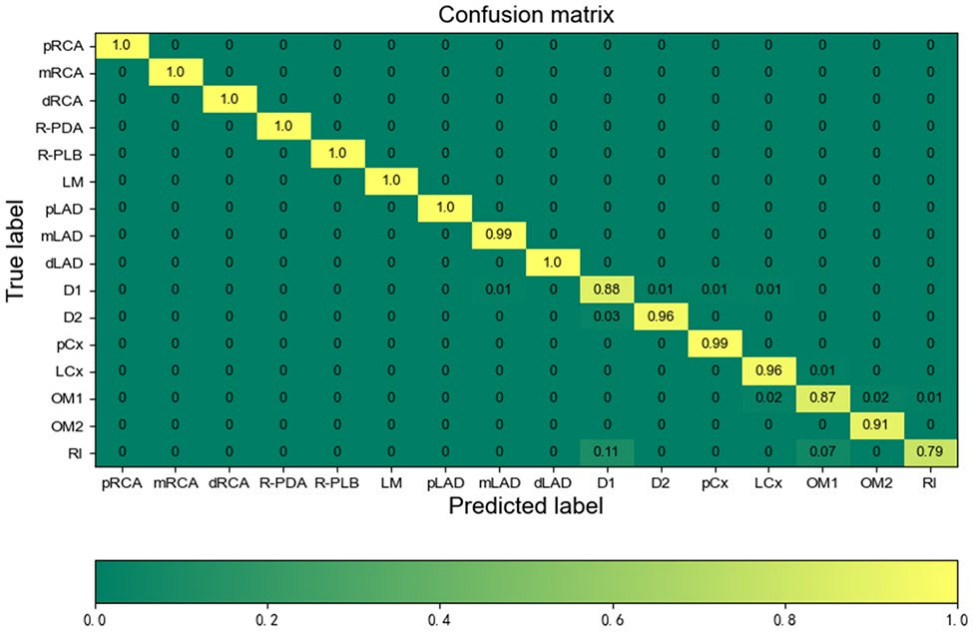
Confusion matrices for the method

**Fig. 6:**
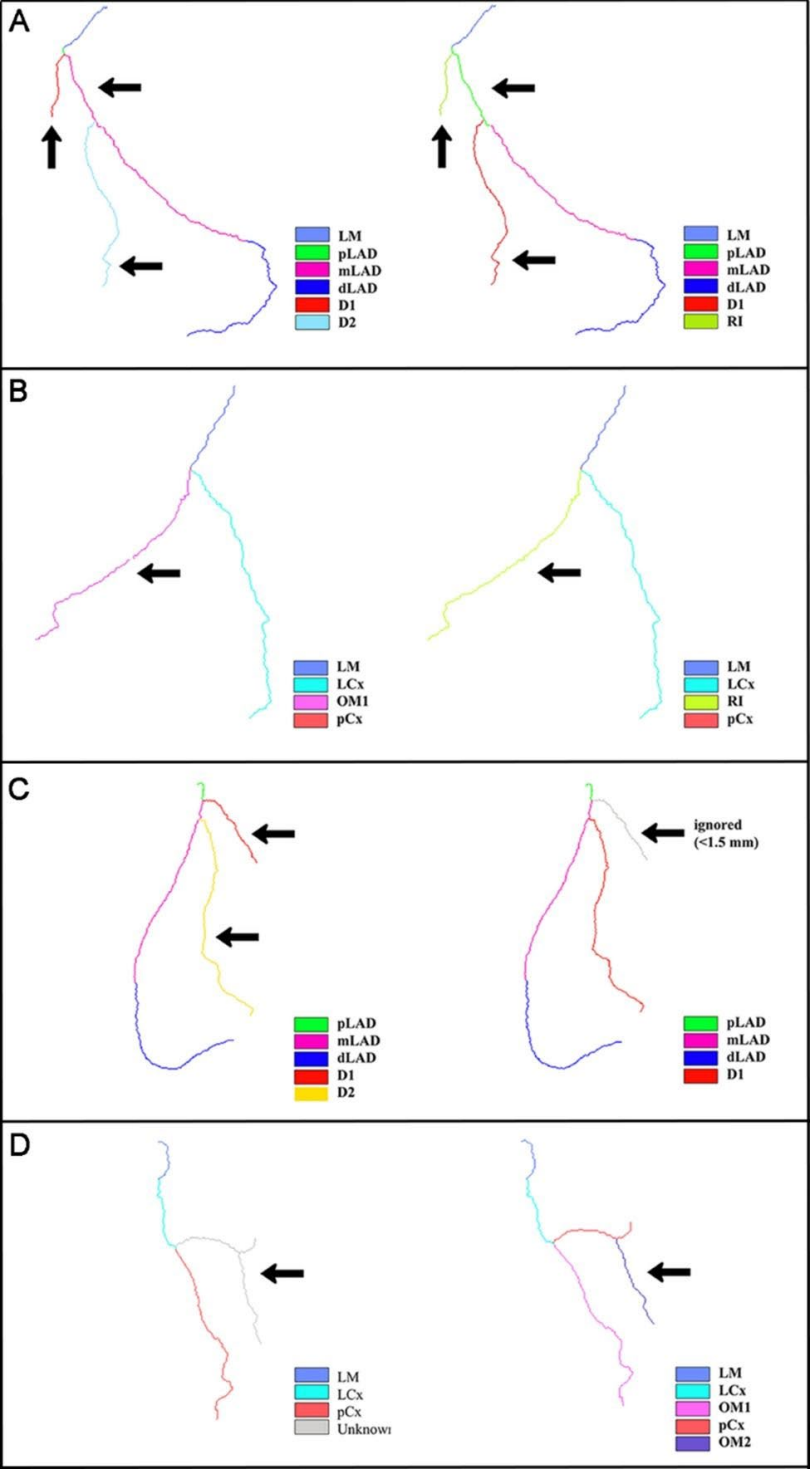
Examples of misclassifications and the correct label. (**A**) The RI was misclassified as the D1; (**B**) The RI was misclassified as the OM1; (**C**) The D2 was misclassified as the D1; (**D**) The OM1 was misclassified as LCx. The left panels show the misclassifications; the right panels show the correct labels

To further analyze misclassified cases, normalized confusion matrices were employed for segments labeled as true positives (TPs) (Fig. 5). Notably, major branches such as mLAD and LCx exhibited low misclassification percentages. In contrast, side branches, including D1, D2, OM1, and RI, displayed relatively higher misclassification percentages. In our proposed method, 3.0% of D2 segments, 2.0% of OM segments, 11.0% of RI segments, and 7.0% of RIs were misclassified as D1, LCx, D1, and OM1, respectively. Further examples of misclassifications are provided in Fig. 6.

When comparing the results between the two experts, we found that the two experts exhibited disagreements in the labeling of 126 (4.7%) segments within the 157 patients. Disagreement percentages for OM1, R-PLB, OM2, D1, D2, and RI labeling were 20.6%, 19.1%, 15.9%, 11.1%, 10.3%, and 9.5%, respectively. Conversely, agreement percentages for pRCA, mRCA, dRCA, R-PDA, LM, pLAD, mLAD, dLAD, pCx, and LCx labeling were 100.0%, 100.0%, 100.0%, 98.1%, 100.0%, 100.0%, 99.4%, 99.4%, 98.1%, and 94.3%, respectively.

## Discussion

In this study, we presented an automatic labeling algorithm designed for coronary artery identification using CCTA images. Initially, we introduced the distance transformation algorithm, highlighting its application in the automatic labeling of coronary arteries. Our algorithm efficiently identified coronary tree segments and assigned accurate labels. We conducted extensive evaluations on a dataset consisting of 157 patients, demonstrating outstanding performance. Notably, our algorithm achieved an impressive overall accuracy of 94.0%, with remarkable results, especially for the four primary arteries.

Across all patients, labels for proximal segments, including pRCA, pLAD, pCx, and LM, consistently appeared. Agreement rates were higher in these segments compared to middle and distal segments. This observation aligns with the findings of Cao et al., who developed 3D models for both right- and left-dominant coronary circulation [7]. However, accuracy was lower for D1, D2, OM1, OM2, RI, and R-PLB segments. This discrepancy often occurred when diagnosing patients with absent or improperly extracted diagonal or marginal branches. Moreover, the agreement proportions for these segments were relatively small, indicating the challenges experts faced when distinguishing D1 from D2 and OM1 from OM2.

In our study, pCx and LCx segments demonstrated higher accuracy (98.7% and 93.0%, respectively) compared to other methods, especially when considering the distance between LCx and the LA and LV [10, 21]. Typically, distal segments within the same branch showed lower overlap compared to proximal and middle segments. In some cases, LCx was incorrectly classified as OM1 or OM2 (1.0%). This misclassification could occur due to missing OM1 labels or incomplete OM1 extraction, leading to incorrect endpoint assignment for pCx [2]. Similarly, the absence of D1 and D2 extraction affected the accuracy of pLAD and dLAD. Incorrect labels or endpoint assignments in one segment could propagate errors to subsequent segments. Despite the anatomical challenges presented by side branches, our method outperformed others, primarily due to the higher accuracy in pCx and LCx labeling [7, 10]. The lower overlap for RI and R-PLB was primarily attributed to difficulties in RI and R-PLB detection. To improve labeling accuracy, additional efforts are required for RI and R-PLB detection. Additionally, variations in start point definitions contributed to the small agreement ratios for OM1, R-PLB, and RI labels.

Comparing our results with other algorithms, Akinyemi et al. utilized a Gaussian classifier based on geometric features of coronary arteries. However, anatomical differences in training datasets could affect label accuracy. In contrast, our method exhibited robustness by utilizing distances to the LA and LV [6]. Additionally, Mehmet et al. proposed a labeling method involving geodesic paths in a standard model of coronary trees [8]. They relied on four-chamber positions to set coronary tree coordinates, while we improved accuracy by using distances between each centerline point and the LA and LV. Moreover, Mehmet et al. did not include RI in their labeling method. Compared to their labeling results (overall accuracy of 86.5% for coronary arteries) for automatically detected centerlines, our algorithm achieved a higher labeling accuracy (94.0%). Fischer et al. employed Tree-LSTM, a recurrent neural network, achieving an impressive average accuracy of 96%, although the LCx accuracy was relatively lower (89.7%) [22]. Overall, our method outperformed others, primarily due to the higher accuracy in pCx and LCx labeling.

In our study, we utilized the U-Net architecture, as described by Çiçek et al., and specifically trained a model for coronary artery segmentation [18]. The U-Net model was trained using methods outlined in our previous study, tailored for coronary artery segmentation, and exhibited excellent performance in this study [20]. Additionally, we employed a distance transformation algorithm to label LCx and pCx segments, achieving accuracies of 99.4% and 93.0%, respectively. However, direct comparisons with other methods are challenging due to differences in principles and datasets used. Variations in data distribution, quality, feature representation, and biases introduced by different datasets can impact method performance and generalizability, making definitive conclusions about relative effectiveness difficult.

This study has several limitations. First, we did not label L-PDA and L-PLB due to their limited consideration in right-dominant diagnostic processes. Second, the performance assessment of the automatic centerline extraction method significantly influenced labeling results. In the future, we will focus on improving artery centerline extraction to enhance labeling accuracy and avoid missed or misclassified arteries. Third, the sample size, particularly for a neural network approach, is relatively small. Multicenter studies with larger sample sizes are warranted to further enhance the method. Future studies will include additional CCTA procedures at our hospital and leverage publicly available datasets to broaden our research scope and increase data diversity, enhancing comparability. Fourth, evaluations in this study were conducted at the segment level rather than the patient level, making it challenging to assess patient-level variability. Future evaluations will consider both segment- and patient-level assessments.

In summary, the labeling algorithm presented in this research offers the potential for precise and entirely automated coronary artery labeling in CCTA images. Moreover, this algorithm is applicable to both pathological and healthy datasets. The integration of this algorithm into coronary artery labeling processes holds promise for significantly reducing the radiologists’ workload in the foreseeable future.

## Data Availability

Data and materials are available upon reasonable request from the corresponding author.
